# Homocysteine inhibits angiogenesis through cytoskeleton remodeling

**DOI:** 10.1042/BSR20170860

**Published:** 2017-09-19

**Authors:** Lemen Pan, Guanfeng Yu, Jingyong Huang, Xiangtao Zheng, Yinghua Xu

**Affiliations:** 1 Department of Vascular Surgery, The First Affiliated Hospital of Wenzhou Medical University, Nanbaixiang, Ouhai District, Wenzhou 325015, Zhejiang Province, P.R. China; 2 Department of Medical Oncology, Sir Run Run Shaw Hospital, College of Medicine, Zhejiang University, No. 3 Qingchun East Road, Hangzhou 310016, Zhejiang Province, P.R. China

**Keywords:** angiogenesis, actin stress fiber, endothelial cells, HUVEC, homocysteine

## Abstract

Homocysteine (Hcy) is an intermediate non-diet amino acid connecting methionine and folate cycles. Elevated total Hcy level in blood, denoted as hyperhomocysteinemia, has emerged as a prevalent and strong risk factor for multiple diseases including atherosclerotic vascular disease in coronary, cerebral, and peripheral vessels. Its detrimental effect on vascular system implies the potential application as an inhibitor of angiogenesis. However, the detailed mechanism is unveiled. Inhibitory effect of Hcy was assessed on vascular endothelial growth factor (VEGF) induced cell proliferation and migration with endothelial cell (EC) culture system. Its effect on angiogenesis was further examined *in vitro* and *in vivo*. After Hcy treatment, key angiogenic factors were measured by RT-qPCR. Cellular skeletal structure was also evaluated by actin stress fiber staining. VEGF-induced human umbilical vein EC (HUVEC) proliferation and migration were dramatically down-regulated by Hcy in a dose-responsive manner. Hcy treatment significantly inhibited the VEGF-induced angiogenesis *in vitro* by tube formation assay and chick chorioallantoic membrane (CAM) vessel formation *in vivo*. Key angiogenic factors like VEGFR1/2 and angiopoietin (Ang)1/2 were substantially reduced by Hcy in HUVEC- and VEGF-induced actin stress fiber cytoskeletal structure was abolished. We demonstrated that Hcy could inhibit angiogenesis by targetting key angiogenic factor and disruption of actin cytoskeleton which is crucial for cell migration.

## Introduction

Homocysteine (Hcy) is a sulphur-containing α-amino acid. It is not found in proteins and cannot be obtained from diet. In cells, it is biosynthesized from methionine via a cycle of chemical reactions. First, methionine is converted into S-adenosylmethionine (SAM) by methionine adenosyltransferase (MAT) in ATP-dependent manner. SAM is a ubiquitous methyl group donor which is required for a large family of SAM-dependent methyltransferases for methylation of DNA, RNA, proteins, and lipids. During the methylation reaction, SAM is then converted into S-adenosylhomocysteine (SAH) after the methyl group is transferred to acceptor molecules. SAH gives rise to Hcy via hydrolysis reaction to remove adenosine. Biochemically, Hcy can be recycled to form methionine by methylation or combined with serine to give rise to cysteine which is the precursor of an important antioxidant factor glutathione. Hcy transsulphuration pathway is critical for Hcy catabolism and is considered as a major source of glutathione in the liver [1–3].

The normal concentration of total Hcy in plasma of adults is approximately 10 μM [4]. Over 90% of total plasma Hcy is bound to plasma proteins, and only traces of free Hcy, approximately 0.1 μM, are present in plasma [5]. Hyperhomocysteinemia, elevation of plasma Hcy level, is caused by the deficiency of dietary intake of vitamins B_12_, B_2_, folate, and choline [6]. In this condition, increased level of Hcy undergoes auto-oxidation of thiol group to generate hydrogen peroxide, and other reactive radical oxygen species which then leads to oxidative stress in cells [7,8]. Increased oxidative stress subsequently causes dysfunction of endothelial cells (ECs), swelling and vacuolization of ECs, fibrin deposition, and even clot formation in vascular vessels. Therefore, Hcy is considered a risk factor for cardiovascular disease [9,10].

On the other hand, Hcy is recently reported to play an important role in angiogenesis [11–15] which is a hallmark of cancer development [16,17]. Angiogenesis by definition is the physiological process in which new blood vessels form from pre-existing ones. It is vital in growth and development, also it is essential for tumor growth which depends on the supply of nutrients, oxygen, and waste disposal. Moreover, migration of tumor cells into distal regions also requires the route of blood vessel, and it has been the primary killing factor for tumor mortality.

Angiogenesis is under strict regulation of cellular microenvironment by the circulating positive and negative signals. The primary angiogenic signal is vascular endothelial growth factor (VEGF) which binds to VEGF receptor found on EC surface and promotes EC growth and migration toward the source of VEGF. Anti-angiogenesis factors have been continuously developed and widely used as antitumor agents, for instance avastin, endostatin, and some VEGF inhibitors such as sorafenib, axitinib, and pazopanib [18]. Since Hcy reduces EC proliferation, which plays a key role in angiogenesis, it has been proposed as an inhibitor of angiogenesis. The Hcy-dependent impairment of angiogenesis is largely caused by the decrease in glutathione peroxidase expression and consequent increase in oxidant stress, leading to endothelial progenitor cell dysfunction [19,20], decreased bioactive nitric oxide generation [7,21], and dysregulation of matrix metalloproteinase (MMP) activity as well as tissue remodeling [22].

The actin cytoskeleton and associated proteins play a critical role in cell–cell adhesion [23]. Through their cytoplasmic tails, junctional adhesion proteins may bind to cytoskeletal and signaling proteins, which allow the anchoring of the adhesion proteins to F-actin and the transfer of intracellular signals inside the cell [24,25]. Actin cytoskeleton is also implicated in angiogenesis [26,27]. In the present study, we examined the function of Hcy as an inhibitor of angiogenesis in EC model and our results showed that Hcy could counteract the proliferative effect of VEGF on EC to suppress the cell migration and tube formation ability *in vitro*. At molecular level, it reduced the mRNA levels of angiogenic factors such as VEGFR1/2, angiopoietin (Ang)1/2 and disrupted the actin stress fiber formation. The inhibitory role of Hcy on angiogenesis was also confirmed in chick chorioallantoic membrane (CAM) assay which was more physiologically relevant.

## Materials and methods

### Cell culture

Human umbilical vein ECs (HUVECs) were purchased from ScienCell (CA, U.S.A.), and the human hepatic epithelial cell line (WRL-68) and human fibroblast-like fetal lung cells (WI-38) were purchased from American Type Culture Collection (ATCC; VA, U.S.A.). HUVECs were cultured in EC medium (ECM; ScienCell, U.S.A.) supplemented with 5% heat-inactivated FBS (ScienCell, U.S.A.), 1% penicillin/streptomycin (ScienCell, U.S.A.) and 1% EC growth supplement (ECGS; ScienCell, U.S.A.). WI-38 and WRL-68 were maintained in Dulbecco’s modified Eagle’s medium (DMEM; Gibco, CA, U.S.A.) and Roswell Park Memorial Institute medium 1640 (RPMI; Gibco, U.S.A.), respectively, supplemented with 10% heat-inactivated FBS (Sigma–Aldrich, MO, U.S.A.) and 1% penicillin/streptomycin (Gibco, U.S.A.). All cells were incubated at 37°C in humidified 5% CO_2_ and 95% air.

### *In vitro* migration and invasion assays

Migration assay was performed by the Boyden chamber method in 24-well plates with inserts of 6.5-mm diameter and 8-μm pore size (Transwell; Corning Inc., NY, U.S.A.). DMEM/F12 containing 10% FBS as a chemoattractant was placed in the lower wells, respectively. In brief, 200 μl of the HUVEC cell culture was added to the upper compartments. The chamber was incubated at 37°C under 5% CO_2_ for 16 h. After incubation, the non-migrating cells were removed from the upper surface of the filter using a cotton swab. The filters were fixed in methanol for 15 min and stained with 0.1% Crystal Violet for 15 min. Migration was quantitated by counting the stained cells that migrated to the bottom side of the membrane using an optical microscope (100× magnification). All experiments were made in duplicate and replicated three times at least.

### Scratch-wound directional migration assay

HUVECs were seeded at a cell density of ×10^5^ cells/well in a 96-well microtiter plate and allowed to grow into a confluent monolayer overnight. Then, the monolayer was scraped using a sterile 20–200 µl micropipette’s pipette tip to create a wound of ±1 mm width. The cells were washed twice with Hanks’ balanced salt solution (HBSS; Sigma–Aldrich, U.S.A.) and replaced with fresh medium containing indicated concentrations of NC. After 8 h, the cells were stained with Hoechst 33342 and Cellomics® whole cell stain green (Thermo Fisher Scientific, Waltham, MA, U.S.A.). Cell migration was estimated by measuring the number of ECs that had migrated from the edge of the wounded monolayer. An area of 512 × 512 pixels from the wounded area was acquired using Cellomics Array Scan HCS Reader and the number of migrated cells was calculated by the HCS automated algorithm. Inhibition of migration was represented by a decrease in the number of cells in the image acquired relative to the untreated control. For each monolayer sample, three measurements were taken for three independent wounds.

### Chick CAM assay

Fertilized white Leghorn eggs were incubated at 37°C in a humidified incubator and windowed. On day 7 of development, sterile filters soaked with either vehicle or VEGF (100 ng/disk) in the presence or absence of NC (9 µg/disk) were applied to relatively avascular regions of the CAM. CAMs were fixed (4% paraformaldehyde in PBS) *in ovo* on day 9 and photographed in the localized area of the filter. The newly capillarized area in the region of each filter was quantitated using Leica QWin Lite software and neovascularization is expressed as an angiogenic index (*n*= 12–15 eggs per treatment). The present study was approved by the Ethics Committee of The First Affiliated Hospital of Wenzhou Medical University.

### RNA extraction and real-time PCR

HUVECs were treated with 7 mM NC for 16 h, PBS was used as a control. Briefly, RNA extraction from subconfluently treated or non-treated cells was performed using 1.0 ml of TRIzol (Invitrogen, Carlsbad, CA, U.S.A.) for 1 × 10^6^ cells according to manufacturer’s recommendations. RNA integrity was assayed by agarose gel electrophoresis and treated with DNAse (RQ1 RNAse free DNAse – Promega, Madison, WI, U.S.A.). cDNA and PCR were performed using SuperScript III Platinum One-step qRT-PCR Systems (Invitrogen, U.S.A.). Gene expression was measured in 7500 Fast (Applied Biosystems, Waltham, MA, U.S.A.) using *GAPDH* (Hs99999905_m1) as an endogenous gene. Taqman gene expression assay from Applied Biosystems were performed for *VEGFR1* and *Ang1/2* genes, respectively.

### Immunocytofluorescence microscopy

The effects of PA on the actin and tubulin cytoskeletal systems of HUVECs were investigated by immunofluorescence. Briefly, HUVECs at ∼80% confluence were treated with PA for 16 h and stained with Phalloidin for F-actin and antipaxillin antibody for paxillin, respectively. Images were acquired on conventional fluorescence microscope and the effects on F-actin and paxillin were analyzed by Morphology BioApplication Algorithm (Thermo Fisher Scientific, U.S.A.).

### Statistical analysis

All values were expressed as means ± S.E.M. The data were analyzed using Student’s *t* test for two-group comparisons or using two-way ANOVA followed by the Tukey’s post hoc tests. GraphPad Prism 7.0 statistical and graphing software was used for the statistical analyses. Differences were considered significant at *P*<0.05.

## Results

### Hcy inhibits EGFP-induced cell proliferation and migration

**Figure 1:**
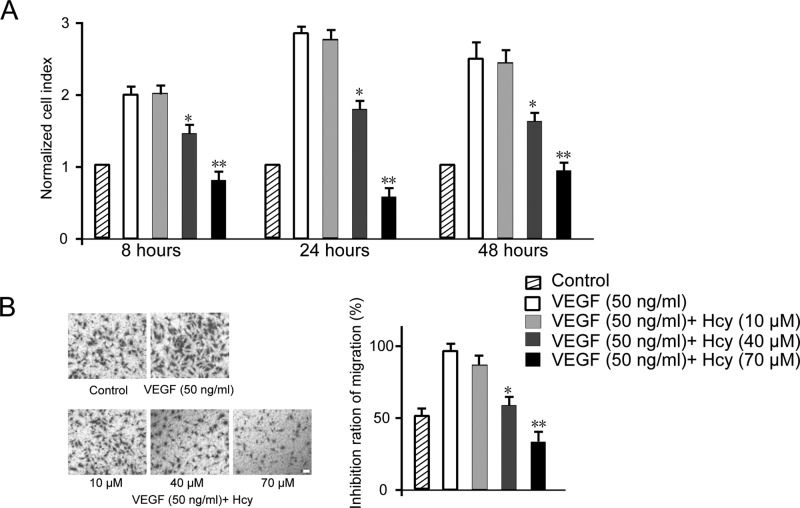
Effects of Hcy on HUVEC proliferation (A) and migration (B) Data are expressed as means ± S.E.M. of three independent experiments. Migrated cells were observed using a modified Boyden chamber assay. The data are presented as percentages of inhibition. Statistical significance is expressed as **, *P*<0.001; *, *P*<0.05 compared with VEGF control (*n*=4). Scale bar indicates 50 µm.

During the angiogenesis process, ECs are stimulated to migrate, proliferate, and invade surrounding tissues to form capillary tubules capable of carrying blood. VEGF is a potent inducer of EC proliferation. Using an established cell model HUVEC, we first examined the effect of Hcy on VEGF-driven cell proliferation assay, with dosage range based on literature and pilot study. As shown in Figure 1A, HUVECs’ growth rate was promoted by VEGF (50 ng/ml) from 8 to 24 h and maintained at steady state from 24 to 48 h. Under different dosages of Hcy, HUVEC growth was monitored. We noticed that 10 μM of Hcy did not make much difference, while 40 μM Hcy showed ∼25% reduction in cell growth. Strikingly, 70 μM Hcy completely abolished the proliferative effect of VEGF at all time points. It is worth noting that Hcy at doses higher than 70 μM could not induce further inhibition in our pilot study (results not shown). Next, we investigated the migration capability of HUVECs under treatment of Hcy with modified Boyden chamber assay. Compared with control condition, VEGF could significantly enhance the migration of HUVEC to the extent of almost two folds. Similarly, although 10 μM Hcy caused negligible effect, 40–70 μM Hcy showed significant inhibitory effect in a dose-responsive manner. As shown in Figure 1B, 40 μM Hcy completely reverted the VEGF effect to the level of control cells and 70 μM Hcy showed 40% suppressive effect further compared with 40 μM Hcy treatment condition. Collectively, our results showed that high concentration Hcy treatment could robustly suppress HUVEC growth and migration.

### Hcy suppresses the *in vitro* angiogenesis

**Figure 2:**
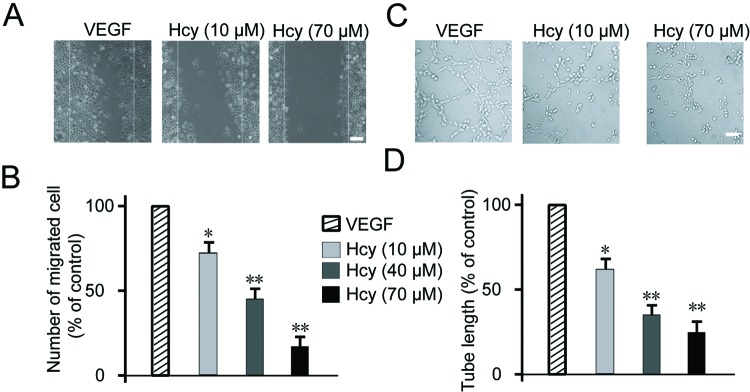
Effects of Hcy on HUVECs migratory ability and tube formation (**A**) Confluent monolayer of HUVECs was wounded and treated with either Hcy (10, 40, 70 μM) or medium alone (untreated control) for 24 h following VEGF stimulation. The cells were then fixed and stained with Hoechst 33342 and Cellomics® whole cell stain green. (**B**) Quantitation of the number of migrated cells after 24 h exposure to indicated concentrations of Hcy. For each monolayer sample, three measurements were taken in three independent wounds. Percentage of inhibition was expressed using untreated wells at 100%. (**C**) Effects of Hcy on tube formation. (**D**) Quantitative data of tube formation after treatment with Hcy for 2 h following VEGF stimulation. Data are expressed as means ± S.E.M. of three independent experiments. Statistical significance is expressed as **, *P*<0.001; *, *P*<0.05 compared with VEGF. Scale bar indicates 50 µm (A)/20 µm (C).

Angiogenesis is normally activated in the wound healing process. We next performed the wound healing assay on HUVEC cell monolayer with different dosages of Hcy in the presence of VEGF. As shown in Figure 2A, VEGF could readily induce cell migration to fill the gap introduced by wound after 24 h, 10 μM Hcy treatment could reduce the cell migration by 25% and 70 μM Hcy completely inhibits VEGF-induced cell migration, as quantitated in Figure 2B. During the angiogenesis, ECs need to protrude from old vessel in a tube format. The ability to form tubing is therefore important and is assessed as a standard method to quantitate the potential of angiogenesis [28]. We then examined the effect of Hcy with tube formation assay. As shown in Figure 2C, VEGF could stimulate the formation of capillary-like tubes on the surface of extracellular matrix, while Hcy treatment showed dose-dependent inhibition of tube formation as quantitated in Figure 2D. Compared with VEGF control, 70 μM Hcy treatment reduced the length of tube by 70%. The above results demonstrated that Hcy could suppress the angiogenesis *in vitro*.

### Hcy impairs angiogenesis *in vivo*

**Figure 3:**
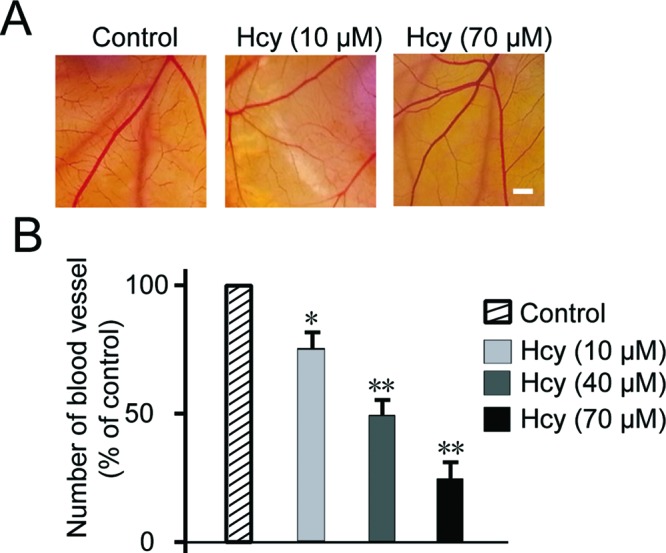
Hcy inhibited angiogenesis *in vivo* Representative images of chick embryonic CAM after treating with Hcy for 48 h (**A**). Quantitative data of chick embryonic CAM after treating with CA4 for 48 h (**B**). Pooled data from 12 to 15 eggs (mean ± S.E.M.). Statistical significance is expressed as **, *P*<0.001; *, *P*<0.05 compared with VEGF. Scale bar indicates 50 µm.

To validate the results in Figure 2, we further evaluated the Hcy effect on angiogenesis in a setting mimicking the *in vivo* situation. CAM assays have been widely used to study angiogenesis and tumor invasion as a robust and cost-effective *in vivo* model. From result in Figure 3A,B, Hcy-treated CAMs clearly showed less capillaries formed from main blood vessel, compared with the control. Quantitation of data from 12 to 15 eggs showed remarkable reduction in blood vessel numbers with dose-responsive effect where 70 μM Hcy showed more than 70% suppression. This result confirmed the *in vitro* data that Hcy treatment could significantly inhibit angiogenesis *in vivo*.

### Hcy represses the angiogenesis factors

**Figure 4:**
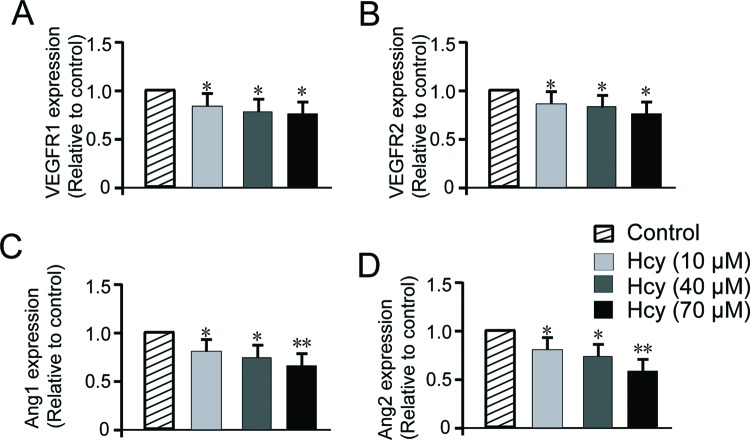
Hcy decreases VEGFR1/2 and Angs (Ang1, Ang2) gene transcription (**A**,**B**) RT-PCR analysis of VEGFR1 and VEGFR2 demonstrated that the treatment with different concentrations of Hcy reduces *VEGFR1/2* mRNA in HUVECs. Data are expressed as fold increase compared with control cells treated with PBS only and are the mean ± S.E.M. of three experiments. Hcy significantly decreased Ang1 (**C**) and Ang2 (**D**) gene expression in a dose-dependent way. The data are representative of three independent experiments performed in triplicate. Statistical significance is expressed as **, *P*<0.001; *, *P*<0.05 compared with untreated control.

To gain more molecular insights into the function of Hcy on angiogenesis, in addition to the reported oxidative stress, we investigated the key angiogenic factors such as VEGFR and Ang in the process. VEGFR1/2 are receptors for VEGF and play important role to convey the signal into cells and regulate gene expression. Angs form dimer or tetramer and bind to receptor Tie2 to activate downstream pathways involved in angiogenesis as well as vascular permeability regulation [29]. HUVECs were treated with various doses of Hcy for 16 h and mRNA levels of VEGFR1/2, Ang1/2 were quantitated by RT-qPCR method. As shown in Figure 4A,B, *VEGFR1/2* mRNA level showed dose-dependent decrease upon Hcy treatment (*P*<0.05) compared with untreated control. *Ang1/2* mRNA showed 20–30% decrease with 10 and 40 μM Hcy (*P*<0.05) and even greater decrease with 70 μM Hcy treatment (*P*<0.01) (Figure 4C,D). These results supported the suppressive role of Hcy on multiple key factors for angiogenesis. This could partially account for the inhibitory effect of Hcy presented in previous figures (Figures 1-3).

### Hcy disrupts the actin stress fiber formation

**Figure 5:**
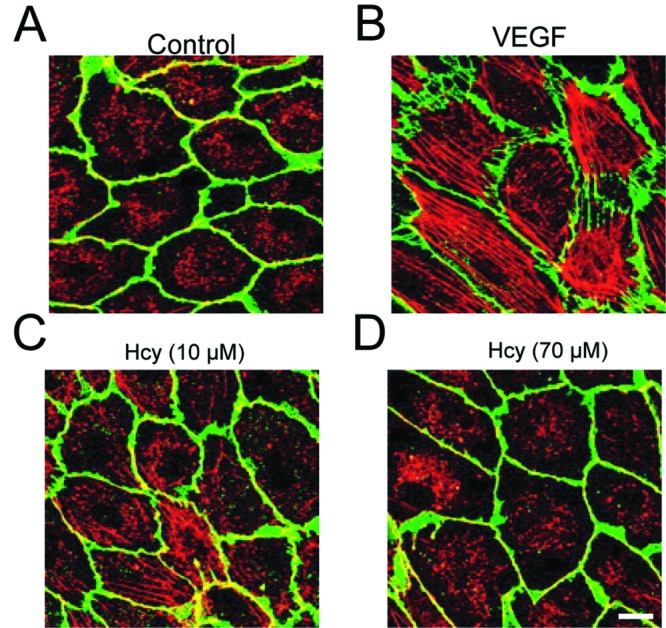
Hcy inhibits VEGF-induced actin stress fibers in HUVECs (**A**) HUVECs were fixed and stained with Phalloidin for F-actin (red), and with Dil for membrane (green), respectively. (**B**–**D**) HUVECs stimulated by 50 ng/ml VEGF were treated with different concentrations of Hcy for 16 h. Scale bar indicates 20 µm.

Stress fibers are contractile actin bundles found in non-muscle cells and are found to play an important role in cellular contractility by providing the mechanic force for multiple functions such as cell adhesion, migration, and morphogenesis [30]. The function of stress fiber in angiogenesis has been elusive. Here, we tested Hcy effect on the formation of stress fiber in HUVECs as another mechanistic explanation for its suppressive role (Figure 5A–D). As shown in Figure 5, VEGF treatment could induce the robust formation of stress fiber inside cells. Interestingly, 10 μM Hcy could dramatically reduce the stress fiber length. Hcy (70 μM) almost completely disrupted the stress fiber structure. Our result added a new layer of mechanism about Hcy-mediated regulation of angiogenesis through perturbation of actin cytoskeleton dynamics and maintenance.

## Discussion

Hcy is an intermediate metabolite connecting the methionine cycle to cysteine biosynthesis. It has been suggested to play a role in oxidative stress, endothelial dysfunction, and acute inflammatory response. However, the function of Hcy in angiogenesis is still under debate. In the present study, we demonstrate the inhibitory effect of Hcy on VEGF-induced EC proliferation, migration. High dose of Hcy could significantly impair angiogenesis *in vitro* and *in vivo*, possibly through down-regulation of key angiogenic factors such as VEGFR and Ang, and disruption of actin stress fiber formation which is crucial for cell motility and morphology. Our results lent support to the potential application of Hcy as an anti-angiogenetic agent in cancer therapy.

One big concern for Hcy as an anti-angiogenesis drug is that high level of Hcy would pose threat to the cardiovascular system as it causes damage to ECs and promotes atherosclerosis and thrombosis. Normal concentration of Hcy in plasma is approximately 10 μM and more than 90% of total plasma Hcy exists as a conjugate with plasma proteins [31]. When the concentration is raised to more than 15 μM in plasma, this will cause a medical condition called hyperhomocysteinemia which is frequently associated with deficiency of B_6_, B_12_ vitamins [32]. There are two types of hyperhomocysteinemia: (i) the rare but severe forms are due to major genetic mutations in the enzymes implicated in Hcy metabolism; (ii) the more common forms cause moderately elevated Hcy levels related to a pathogenesis such as genetic and environmental factors. When the level of Hcy is between 16 and 30 μmol/l, it is classified as moderate, 31–100 μmol/l is considered intermediate, and a value above 100 μmol/l is classified as severe hyperhomocysteinemia [33,34]. The concentration we tested to be effective to inhibit angiogenesis fell into the range of intermediate hyperhomocysteinemia. In fact, large-scale meta-analysis concludes that elevated Hcy is just a modest independent risk factor for ischemic heart disease and stroke incidence [35]. Therefore, the value of Hcy as an angiogenesis inhibitor could be explored more thoroughly in the clinical setting of tumor therapy since the inhibitory effect of Hcy is relatively rapid and the advantage for cancer patient will overcompensate the moderate risk.

In our study, we first tested the effect of Hcy on basic characteristics of EC growth and reconfirmed its inhibitory function on cell proliferation and migration (Figure 1). We used HUVECs as EC model which has been widely accepted in the field since its report by Rhim et al. [36]. Our result was consistent with previous studies on Hcy with different cell lines HMEC1 [13], ECV304 [37], and tumor cell line [38]. ECs play important role in forming new vessels in tube-like structure and sprouting out from old vessels during angiogenesis. Therefore, we validated the effect of Hcy on angiogenesis *in vitro* by tube formation assay or *in vivo* by CAM assay. Both these results demonstrated the dramatic inhibition of angiogenesis (Figures 2 and 3), which is also well supported by multiple studies [12–14].

The mechanisms of Hcy-induced EC dysfunction and inhibition of angiogenesis have been mainly attributed to the disruption of antioxidant glutathione production which subsequently leads to ROS formation and oxidative stress in the ECs as well as other types of target cells [39–42]. Hyperhomocysteinemia increases oxidative stress and is closely related to accumulation of asymmetric dimethylarginine (ADMA), an endogenous nitric oxide (NO) synthase (NOS) inhibitor that inhibits the activity of endothelial NOS (eNOS) and inducible NOS (iNOS) which play crucial role in cardiovascular regulation [43–45]. However, many of these studies used Hcy concentrations far beyond physiological range (1–10 mmol/l). Under such conditions, it may lead to the generation of reactive oxygen species in the absence of *in vivo* antioxidant defense systems [46–48]. Indeed, there was some discrepancy regarding the role of Hcy in the promotion of oxidative stress [49], although it was still favored by the mainstream researchers that ROS plays an important role in Hcy-induced endothelial dysfunction. In our study, we chose to tackle this problem from another aspect since there have been a plethora of studies supporting the oxidative stress theory. In addition to the impaired EC proliferation by ROS stress, ECs were exposed to complex regulatory network of cytokine and growth factor such as VEGF and its receptor-mediated signaling events, Ang and Tie2 receptor signaling, amongst many others. Interestingly, our result suggested that Hcy had suppressive function on those signaling factors in a dose-dependent manner (Figure 4). Our finding about Hcy and VEGF signaling pathway was supported by other researchers [15,50]. The connection between Hcy and Ang was first time reported in the field by us, which sheds more light on the mechanism of Hcy-mediated angiogenesis regulation. Nonetheless, we had to point out that the effect of Hcy on those angiogenic signaling factors was not so dramatic as what we observed on the phenotypic effect of Hcy on VEGF-induced angiogenesis and cell proliferation (Figures 1-3). This difference suggested that the regulation on cytokine signaling, particularly the mentioned ones, was not the primary player in the Hcy effect on angiogenesis which was supposed to be multifaceted mechanisms.

We continued to explore some new mechanisms shown in Figure 5 that Hcy disrupted the VEGF-induced actin stress fiber formation. It has been reported that VEGF can promote actin remodeling and cell migration through Rho and ROCK signaling. However, it was relatively lack of attention for the role of Hcy in angiogenesis, so far only Sen et al. [51] briefly mentioned that Hcy and cyclic stretch combined together to regulate endothelial focal adhesion protein redistribution and cell remodeling. Our study pointed out that cytoskeletal dysregulation could be the new dimension of mechanism regarding Hcy-induced angiogenic suppression. The detailed signaling pathway about how elevated Hcy suppresses VEGF-induced cytoskeletal remodeling will be investigated in future.

## Conclusion

In summary, Hcy was demonstrated as a potent anti-angiogenic factor in our study. It could inhibit VEGF-induced HUVEC cell growth and migration in a dose-responsive manner. Further we confirmed moderately higher concentration of Hcy (70 μM) could suppress angiogenesis process *in vitro* and *in vivo*. The molecular mechanism of Hcy-mediated inhibition was partly accounted by its ability to down-regulate key angiogenic factors like VEGFR and Ang, in addition to the well-established disruption of redox balance in cells. More importantly, we unveiled the unappreciated function of Hcy to abolish VEGF-induced actin stress fiber formation and cytoskeletal remodeling in ECs.

## Abbreviations

Ang: angiopoietin
CAM: chorioallantoic membrane
DMEM: Dulbecco’s modified Eagle’s medium
EC: endothelial cell
Hcy: homocysteine
HUVEC: human umbilical vein EC
NOS: nitric oxide synthase
SAH: S-adenosylhomocysteine
SAM: S-adenosylmethionine
VEGF: vascular endothelial growth factor
